# Rapid and Efficient DNA Extraction Protocol from Peruvian Native Cotton (*Gossypium barbadense* L.) Lambayeque, Peru

**DOI:** 10.3390/mps8030050

**Published:** 2025-05-07

**Authors:** Luis Miguel Serquén Lopez, Herry Lloclla Gonzales, Wilmer Enrique Vidaurre Garcia, Ricardo Leonidas de Jesus Velez Chicoma, Mendoza Cornejo Greta

**Affiliations:** Research Center, Professional School of Environmental Engineering, César Vallejo University, Lambayeque 13001, Peru; lserquenl@ucv.edu.pe (L.M.S.L.); wvidaurre@ucv.edu.pe (W.E.V.G.); rvelez@ucv.edu.pe (R.L.d.J.V.C.); gmendozaco@unprg.edu.pe (M.C.G.)

**Keywords:** native cotton, gossypium, extraction DNA

## Abstract

Efficient extraction of high-quality DNA from plants is a critical challenge in molecular research, especially in species such as *Gossypium barbadense* L., native to Peru, due to the presence of inhibitors such as polysaccharides and phenolic compounds. This study presents a modified CTAB-based protocol with silica columns that is designed to overcome these limitations without the need for liquid nitrogen or expensive reagents. Native cotton samples were collected in Lambayeque, Peru, and processed using a simplified procedure that optimizes the purity and concentration of the extracted DNA. Eight cultivars of *G. barbadense* L. with colored fibers (cream, fifo, light brown, dark brown, orange-brown, reddish, fine reddish, and white) were evaluated, yielding DNA with A260/A280 ratios between 2.14 and 2.19 and A260/A230 ratios between 1.8 and 3.14; these values are higher than those obtained with the classical CTAB method. DNA quality was validated by PCR amplification using ISSR and RAPD molecular markers, which yielded clear and well-defined banding patterns. Furthermore, the extracted DNA was suitable for advanced applications, such as Sanger sequencing, by which high-quality electropherograms were obtained. The results demonstrate that the proposed protocol is an efficient, economical, and adaptable alternative for laboratories with limited resources, allowing the extraction of high-quality DNA from *Gossypium barbadense* L. and other plant species. This simplified approach facilitates the development of genetic and biotechnological research, contributing to the knowledge and valorization of the genetic resources of Peruvian native cotton.

## 1. Introduction

Bioprospecting in plants is a very important field of research due to the great variety of chemical compounds plants contain, many with potential applications in sectors such as the pharmaceutical industry, biotechnology, and other areas. The systematic search for natural substances with the intent of exploiting their commercial value allows the development of new medicines, improved food products, and textile materials, among other resources [1].

To identify the genes and metabolic pathways that give a species biotechnological potential, it is essential to perform an exhaustive analysis of the genetic material (DNA) of the species in question. This analysis requires the use of molecular methodologies and techniques, such as genomic sequencing and polymerase chain reaction, which require high-quality genetic material.

To obtain high-quality DNA, it is essential to use appropriate extraction and purification protocols that maintain the integrity and purity of the DNA. Obtaining good-quality genetic material can be a challenge due to the presence of various inhibitors such as polysaccharides, polyphenols, and other secondary compounds, which is why it is important to develop DNA-extraction protocols that can be used to reveal the biotechnological potential of the species being investigated.

Later molecular techniques evolved towards nucleic acid amplification via polymerase chain reaction (PCR), which allowed the evaluation of different markers such as RAPD, SSR, ISSR, SCAR, and TILLING. This approach reduced the required DNA-extraction volumes to 2 mL, although it came with the demand for higher-quality genetic material. In parallel, sequencing techniques advanced from the first-generation approaches based on the Sanger technique to second-generation techniques based on amplification. Currently, third-generation sequencing techniques such as Nanopore and PacBio are available and have been applied to various plant species [2].

Classical DNA-extraction protocols for plants involve steps such as cell lysis, protein precipitation, and purification. These manual methods can be laborious and are prone to human error, which could affect the efficiency and purity of the extracted DNA, therefore special reagents are required for their implementation. The classical techniques applied to cell lysate include extraction with the use of a saline buffer with CTAB (cetyl trimethylammonium bromide) [3], followed by precipitation with alcohols [4]. Other protocols include those based on SDS (sodium dodecyl sulfate), which are effective for the extraction of DNA from plants with high contents of polysaccharides and phenolic compounds [5]. During DNA extraction, the precipitation stage is crucial to the isolation and purification of the extracted DNA. At this stage, classical solution-based protocols use various alcohols such as isopropanol, isoamyl alcohol, phenol, chloroform, and ethanol [6].

Spectrophotometry is a key tool in this process, as it allows estimation of the DNA concentration by measuring the absorbance at 260 nm and assessing its purity through the A260/A280 and A260/A230 ratios. The A260/A280 ratio is used to assess DNA purity with respect to protein contamination, with a value around 1.8 being considered indicative of pure DNA, while the A260/A230 ratio assesses purity with respect to contaminants such as polysaccharides and phenols, with a value close to 2.0 being ideal. In addition, agarose gel electrophoresis provides information on DNA integrity, with intense and well-defined bands indicating high purity. Restriction enzyme digestion, such as HindIII, is used to assess DNA quality by observation of digested products on an agarose gel.

DNA extraction from plants involves various challenges such as the presence of PCR inhibitors, especially in plant samples rich in secondary metabolites; these inhibitors can compromise the quality and quantity of the extracted DNA and interfere with downstream molecular applications. Among the main inhibitors are phenolic compounds such as polyphenols, which are particularly problematic in plants with high levels of secondary metabolites [7,8]. These compounds have the ability to oxidize and form complexes with DNA, which not only makes DNA extraction difficult, but can also degrade the genetic material. On the other hand, polysaccharides, abundant in plant tissues, tend to co-precipitate with DNA during the extraction process, forming a viscous gel that complicates sample handling and can inhibit crucial enzymes in PCR. Furthermore, protein contamination during DNA extraction not only affects sample purity but can also interfere with polymerase activity and other essential reagents in PCR. These combined factors not only hinder the extraction of high-quality DNA but can also lead to false-negative results or inefficient amplification in PCR, compromising the reliability of subsequent genetic analyses [9,10,11].

A method for extracting DNA from plant tissues that has gained popularity in recent years is the CTAB method; however, this method has been modified to suit research needs. Modifications include the addition of regenerated silica columns to the CTAB extraction method, which allows the effective removal of contaminants. Loading raw DNA onto the silica columns yields high-purity DNA suitable for downstream applications, optimizing both time and process costs [12]. Similarly, the CTAB method has been used with spin columns, in which the action of centrifugal force allows the purification of nucleic acids [13,14].

Additionally, other modifications to the CTAB method have been developed to remove the large amounts of polysaccharides and polyphenols present in plants. To this end, a protocol was optimized that involves the addition of antioxidant compounds such as polyvinyl pyrrolidone (PVP), 1,4-dithiothreitol (DTT), and 2-mercaptoethanol to the CTAB extraction buffer and yields higher-quality amplifiable DNA [12,15].

These advances in DNA-extraction methods reflect a continuous effort to improve the accuracy and reliability of PCR techniques, thus allowing a better understanding and exploration of genetic resources.

The definition of native cotton in the context of Peruvian biodiversity focuses on the species *Gossypium barbadense* L., which is a subspontaneous growing plant of the shrub type with a long life cycle that is commonly found on the edges of farms and by fences, orchards, and roads; it is also cultivated as an ornamental plant in gardens. This species includes renowned commercial cultivars such as Tangüis, Pima and IPA; these are highly valued for the quality of their fiber, which stands out for its softness and resistance. These qualities have led to their export and extensive use in the global textile industry [16]. On the northern coast of Peru, the *Gossypium* genus also includes *G. hirsutum* L., which is cultivated mainly as the commercial cultivar Del Cerro and as an adopted green-fiber cultivar. The latter is notable for its adaptability to diverse climatic conditions and its ability to grow on less fertile soils, highlighting its genetic diversity and potential for agricultural improvement [17].

Research on the native-colored cotton *Gossypium barbadense L*. has advanced significantly through the application of molecular tests that have allowed detailed genetic characterization and a better understanding of its genetic diversity. Recent studies have used a variety of molecular markers, such as RAPDs, RFLP, ISSR, and SSR, to assess the genetic variability of this species in different regions of the world, which has facilitated the identification of promising accessions for genetics-based improvement programs [18,19,20,21]. In the context of Peru, considerable effort has been made to characterize the pigmentation of native cotton; studies have revealed that it depends mainly on the presence and concentration of flavonoids, and genetic barcoding has been used with markers such as matK, ITS2 and rubisco, although these do not always provide an identification at the species or genus level [22].

Linkage maps based on these markers have also been developed, allowing for a deeper analysis of the genetic structure and variability within *Gossypium barbadense* L. populations on the northern coast of Peru, where significant concentrations of this species has been found in 71% of the districts evaluated [23,24]. Genetic evaluation has also included diversity studies in native Amazonian communities, where the *Gossypium barbadense* var. *Brasilensis* variant has been identified as the most prevalent and high genetic variability has been observed; this variability is crucial for the conservation of genotypes and adaptation to changing environmental conditions [24]

There is no universal protocol suitable for all plant species and all types of plant material, so additional modifications are often required. Although several studies have been developed to obtain quality genetic material from the *Gossypium* genus, only a few articles have focused specifically on analyzing the genome of *Gossypium barbadense*. This species has presented technical barriers to obtaining quality DNA due to the presence of inhibitors, which are even more difficult to remove from samples of wild or recalcitrant plants [25]. Several protocols are used with the aim of obtaining quality DNA; they are effective, but they require special reagents. For example, those based on the CTAB method with some modifications often require liquid nitrogen [7,8]. Uncommon reagents such as SLS (sodium lauryl sarcosinate) and LiCl (lithium chloride) are also used [26]. Other genomic studies in Gossypium barbadense employ kits for plant DNA extraction, such as the DNeasy Plant Mini Kit from Qiagen [27,28] or the Bionano Plant Tissue DNA isolation kit from Bionano Genomics. Such kits are often expensive or difficult to import to some countries [28]. This diversity in methods reflects the complexity and variability of the approaches needed to obtain quality DNA from different plants.

Although protocols combining the classic CTAB method with the use of silica columns have been previously reported [29], these methods require heating to 80 °C and the use of liquid nitrogen, which can complicate their implementation, depending on the availability of equipment and laboratory conditions. In contrast, the present work presents a simplified methodology that omits these steps, facilitating its application in resource-limited environments, especially considering that the use of silica columns has become widespread as a result of the COVID-19 pandemic.

## 2. Experimental Design

### Biological Material

Native cotton plants from the Cesar Vallejo University arboretum were used. These plants produce various colors fibers called: cream, fifo, light brown, dark brown, or-ange-brown, reddish, fine Colorado, and white (Table 1).

## 3. Procedure

### 3.1. Collection of Leaf Material

Samples of new leaves were collected 7 days after pruning. Next, the material was washed using distilled water and cuts of ~0.5 cm in diameter were made, avoiding the nervures of the leaves, subsequently 3 cuts per sample were then added to a mortar.

### 3.2. Extraction of Genetic Material

Seven native cotton samples were extracted with the CTAB protocol and silica columns (DNA extraction kit—GENEAID) proposed in this article, using the following steps [12]:

1. In a mortar, place a sample ~0.5 cm in diameter of fresh, young leaves without veins, after add 400 µL of extraction buffer 01, 400 µL of extraction buffer 02 (described in the Table 2).
2. Add 4 µL 2-Mercapto Ethanol and triturate until a homogeneous liquid is obtained.
3. Transfer the mixture to a 2.0 mL tube containing 600 µL of isopropanol and shake.
4. Transfer to a column with a silica filter, place the collection cap and centrifuge at 12,000× *g* for 1 min.
5. Remove the liquid from the cap, and recapping the column.
6. Add 500 µL of Wash 1 and centrifuge at 12,000× *g* for 1 min and remove the liquid from the cap and recapping.
7. Add 500 µL of Wash 2 and centrifuge at 12,000× *g* for 1 min and remove the liquid from the cap and recapping.
8. Centrifuge at a maximum speed and remove the collection cap.
9. Transfer the column silica to a new 1.5 mL tube.
10. Add 100 µL of PCR water and incubate for 5 min at 25 °C.
11. Centrifuge at 12,000× *g* for 1 min and remove the column.

Store the extracted DNA in a refrigerator or freezer.

### 3.3. Quantitative Analysis of Isolated DNA

The isolated DNA was subjected to spectrophotometric analysis using a NanoDrop ND 2000, which allowed determination of the UV absorption ratios at A260/280 and A260/230. These values are indicative of the purity of the DNA, and it is desirable that they be within specific ranges to ensure the quality of the genetic material.

### 3.4. Amplification with ISSR and RAPDs

To determine the quality of the extracted DNA, amplification was performed on random markers with genome-wide coverage: ISSR and RAPD (OPD-12: CACCGTATCC; OPC-15 GACGGATCAG; OPC-19 GTTGCCAGCC), (ISSR-10: (GAGA) 4 GAAT). The PCR master mix consisted of 5× Buffer diluted to 1×, MgCl_2_ at 1.5 mM as an essential cofactor, dNTPs at 0.2 mM, and primers at 1.6 µM to amplify random fragments. Platinum Taq was added at 5 U/µL to catalyze the reaction, and 3 µL of DNA was added as a template. The total volume of the final reaction mix was adjusted to 12.5 µL with water.

### 3.5. Amplification of Specific Genes

An amplification using specific markers was performed in a final reaction volume of 12.5 µL; for this, a Master mix was prepared using 2.5 µL of 5× Buffer, diluted to a final concentration of 1×; also, 0.75 µL of MgCl_2_ (25 mM) was added to a final concentration of 1.5 mM; which will act as an essential cofactor and contribute to the stability of the DNA strand during the process. To supply the necessary nucleotides, 0.25 µL of dNTPs at 10 mM were added to a final concentration of 0.2 mM. Likewise, 0.5 µL of forward primer and 0.5 µL of regression primer were added, both at 10 µM (mentioned in the Table 3). This concentration was used to optimize the efficiency of the amplification and minimize the formation of artifacts. The reaction was catalyzed by 0.2 µL of PROMEGA Taq AT 5 U/µL, ensuring accurate amplification of the DNA fragments, Finally, the volume was completed with 4.8 µL of water, and 3 µL of the extracted DNA sample was added as a template.

Gradient temperatures were set as follows: 94 °C for 10 min for denaturation, 52 °C for 30 min for hybridization or annealing, and 72 °C for 45 min for the final extension or elongation step, all for a total of cycles.

### 3.6. Gel Electrophoresis

Agarose gel electrophoresis was performed with a horizontal electrophoresis chamber and power supply (BioRad, Berkeley, CA, USA), a molecular-grade agarose gel (PROMEGA Promega, Madison, WI, USA), and 1× TAE buffer (PROMEGA Promega, Madison, WI, USA) with an initial voltage of 80 volts for 10 min followed by a voltage of 160 volts for 50 min. Subsequently, a molecular weight marker (100 bp DNA ladder) was added, the gel was stained with ethidium bromide; for this, 6x loading buffer containing parafilm was used to mix the bromide with water, and finally, a UV transilluminator was used to visualize the DNA bands in the agarose gel.

### 3.7. Sequencing

The samples were sequenced through the company Macrogen. The primers used in the specific amplification were used for both the forward and reverse sequences. The DNA sent was processed by Sanger sequencing.

### 3.8. Materials and Equipment

#### 3.8.1. Materials

- Microtube, 1.5 mL
- Cotton leaves.
- Silica column (Geneaid Biotech Ltd., New Taipei City, Taiwan)
- Cetyltrimethylammonium bromide CTAB (EMD Millipore Crop, Burlington MA, USA).
- Ethylenediaminetetra-acetic acid EDTA (Solutest Peru, Lince, Lima, Peru)
- Tris Cl (Promega, Madison, WI, USA)
- Mercaptoethanol (Thermo Fisher Scientific, Waltham, MA, USA)
- Isoamyl alcohol (Promega, Madison, WI, USA)
- Buffer wash 1 (Jiangsu Cowin Biotch. CO., Ltd., CWBIO, Taizhou, China)
- Buffer wash 2 (Jiangsu Cowin Biotch. CO., Ltd., CWBIO, Taizhou, China)
- Agarose (IBI Scientific, Dubuque, IA, USA)

#### 3.8.2. Equipment

- Micropipettes p5 µL, p10 µL, p200 µL, p1000 µL
- 1.5 mL microtube racks
- Microcentrifuge (Scilogex SCI-12, Connecticut, C-6, Rocky Hill, CT, USA)
- Thermal cycler (Bioer Technology, Hangzhou, Zhejiang, China)
- NanoDrop One (Thermo Fisher Scientific, Waltham, MA, USA)

## 4. Expected Results

**Figure 1:**
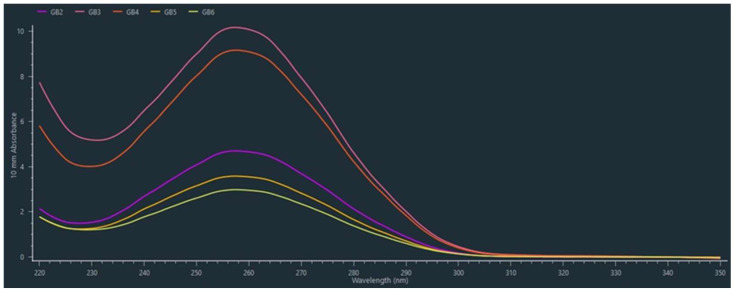
The curves obtained from NanoDrop One (Thermo Fisher Scientific, USA).

The classical CTAB method used by [3] was shown to produce a different range of DNA concentrations compared to our modified protocol and to produce samples of lower purity in most cases. In general, DNA samples extracted by the original CTAB method showed an A260/280 ratio below 1.8, whereas samples extracted by our protocol presented A260/280 ratios ranging from 2.08 to 2.23. Furthermore, the quality of the total DNA extracted by our protocol was assessed by electrophoretic separation, which revealed intense bands in close proximity to the gel wells. In contrast, genomic DNA extracted by the CTAB method [3] did not produce distinct or intact bands. Finally, measurement on the NanoDrop spectrophotometer revealed a single absorbance peak at 260 nm (Figure 1) in the DNA samples extracted by our standardized protocol. These results support the efficacy and reliability of our modified method for DNA extraction from different plant species (mentioned in the Table 4)

**Figure 2:**
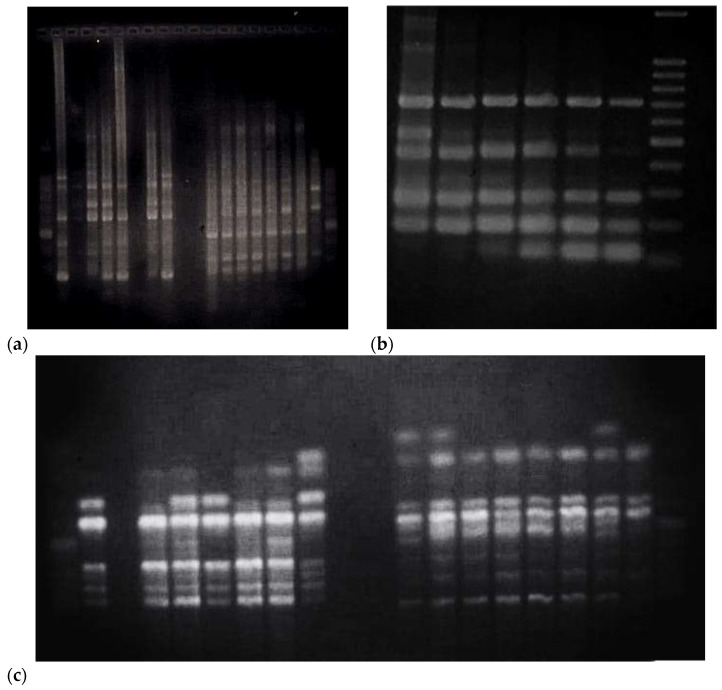
PCR amplification using the RAPD primers OPD-12 (**a**), OPC-15 (**b**), OPC-19 (**c**) and ISSR-10 yielded clear and well-differentiated banding patterns in the DNA samples extracted using the DNA-extraction protocol proposed in this study. This result suggests that the DNA obtained is of high quality and purity, which is essential for obtaining successful amplifications by PCR.

**Figure 3:**
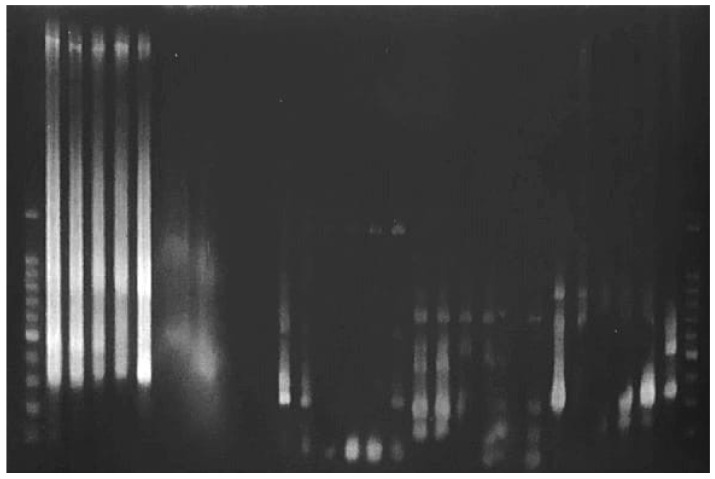
Amplification with RAPD and ISSR markers of DNA extracted using the classical CTAB protocol from native cotton.

**Figure 4:**
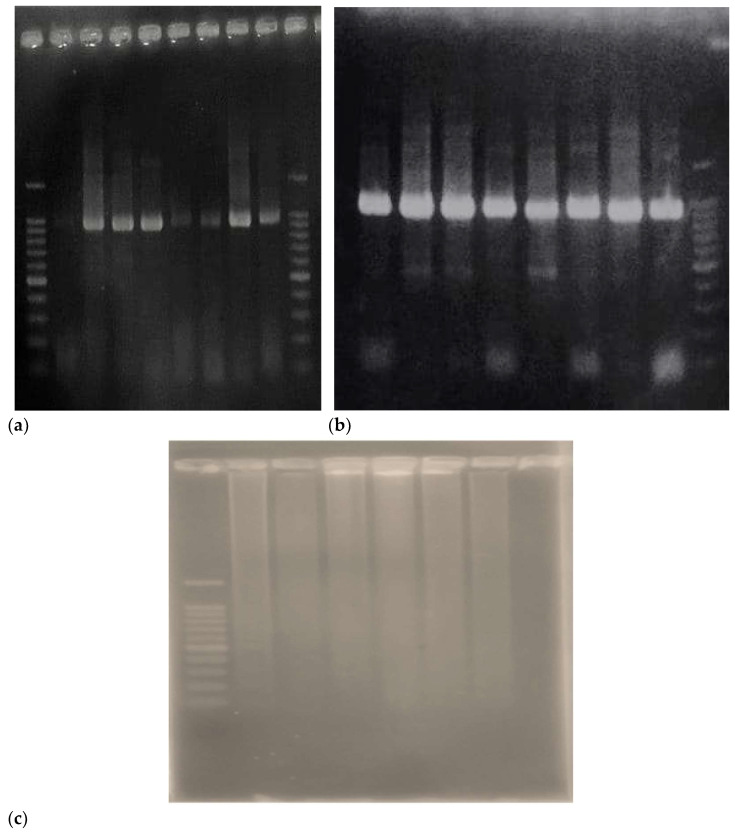
2% agarose gel electrophoresis of the LAR gene 928 bp (**a**) and ANR gene 896 (**b**) and (**c**) LAR amplification with native cotton accessions extracted with the classical CTAB protocol.

In contrast, genomic DNA extracted by the conventional CTAB method from the same native cotton samples presented significant difficulties during amplification (Figure 2 and Figure 3) The bands obtained were weak and poorly defined, indicating that the quality of the extracted DNA might not be suitable for downstream applications such as genetic analysis or identification of molecular markers (Figure 4).

**Figure 5:**
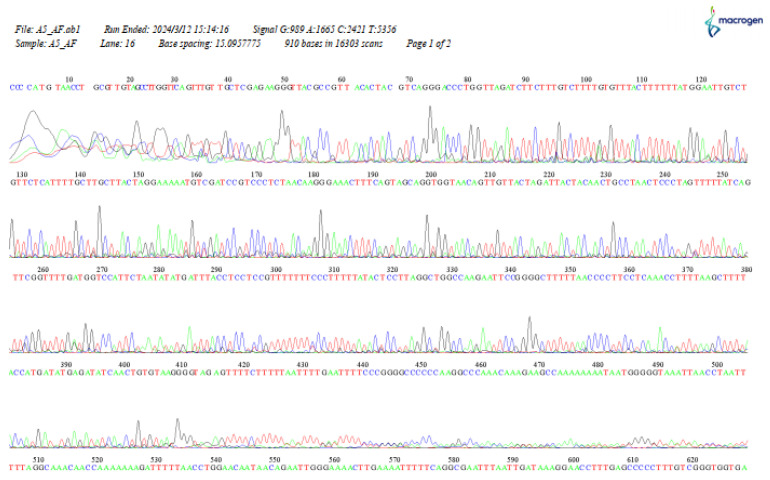
Chromatogram from sequencing of a sample of native cotton.

Sanger sequencing by Macrogen using amplification products of specific ANR and LAR sequences confirmed the observed quality and intensity of the sequences. The resulting curves were well-defined, without the presence of background noise that would make reading the electropherograms difficult, indicating good-quality sequencing (Figure 5).

## 5. Discussion

The efficient extraction of genetic material from plants in the genus *Gossypium* has been the subject of various studies that have evaluated the purity of DNA obtained from different protocols used. The result ranged from 2.14 ± 0.1 A260/280 y 2.6 ± 1.2 A260/230, these A260/280 ratio values have been compared with those obtained using liquid nitrogen protocols (1.87 ± 0.08 for the A260/280), although the A260/230 ratio values are different compared to those obtained using liquid nitrogen (2.20 ± 0.05 A260/230) [14].

Methods based on liquid nitrogen-modified CTAB have proven effective, yielding A260/280 ratios of 2.08 to 2.3 and A260/230 ratios of 1.40 to 2.12 [25,29]. Additionally, the incorporation of lithium chloride has emerged as a promising alternative for obtaining high-quality differential RNA from CTAB- and Triton X-100-based extractions [25,31].

In the search for more accessible methodologies, protocols have been developed that do not use liquid nitrogen, although these require specialized equipment such as refrigerated centrifuges to maintain samples at 4 °C, which represents a significant limitation for laboratories in developing countries. Similarly, the protocol by Ferdous et al. avoids the use of liquid nitrogen but requires temperatures of 67° and freezing [32].

Alternatively, some researchers have proposed methods that avoid both the use of enzymes such as proteinase K and incubations at high temperatures, opting for combinations of CTAB, 2-mercaptoethanol, and PVP, with silica columns used after successive washes and three-phase precipitation; but this involves a greater number of washes, extending the duration of the protocol [33].

The methodology developed by [34] represents a significant advance in nucleic acid-extraction protocols. It involves an optimized combination of CTAB, ÿ-mercaptoethanol, and PVP, dispensing with the use of liquid nitrogen, although it does require maintaining high temperatures and thus requires additional equipment. It also incorporates a purification strategy using silica columns after successive washes and triphasic precipitation with chloroform and alcohols, yielding high-quality genetic material from reduced quantities of plant tissue (15–100 mg). The effectiveness of the method is evidenced by its ability to simultaneously isolate DNA and RNA of sufficient quality for advanced molecular applications such as next-generation sequencing (NGS) and quantitative PCR (qPCR), overcoming the limitations traditionally associated with conventional extraction methods in both monocots and dicots [34].

The effectiveness of these protocols has been validated by using molecular markers such as RAPD and ISSR, which have demonstrated satisfactory amplification capacity via the appearance of clear, sharp bands [21].

Optimal DNA extractions allow for Sanger sequencing where the resulting electropherograms show well-defined curves and low background noise, indicative of high-sequencing quality [35]. This aspect is particularly relevant, as it shows that the proposed method, despite dispensing with expensive or difficult-to-access reagents such as liquid nitrogen or cesium chloride [36], manages to yield genetic material of sufficient quality for advanced molecular applications.

## 6. Conclusions

The combination of CTAB and silica columns, although traditionally associated with the use of liquid nitrogen and phenol-chloroform-isoamyl alcohol and the need for numerous steps, has proven to be effective in this simplified methodology. The methodology does not require low or high temperatures and provides a viable and economical alternative for laboratories with limited resources.

Multiple protocols have been tested to efficiently extract genetic material from plants of the genus *Gossypium*, obtaining results in A260/280 and A260/230 ratios of 1.9 ± 0.1 (A260/280) and 2.0 ± 0.2 (A260/230). Similar results have also been reported using liquid nitrogen within the protocols as detailed (1.87 ± 0.08, A260/280 and 2.20 ± 0.05, A260/230); therefore, it is recommended that the A260/230 ratio should range from 2.0 to 2.22.

Similarly, a protocol that avoids using liquid nitrogen yields ratios that vary between 1.13 and 1.9 (A260/280) but uses a refrigerated centrifuge to keep the samples at 4 °C; this is expensive equipment that many laboratories in developing countries do not have. It avoids the use of liquid nitrogen by using temperatures of 67° and freezing. In the proposed protocol, enzymes such as proteinase K are not used, and no incubations were carried out at temperatures higher than ambient temperatures.

Other protocols with CTAB use cesium chloride and other reagents. A 1994 publication reported a protocol without liquid nitrogen, adding CTAB, B-mercaptoethanol, and polyvinylpyrrolidone (PVP); although these authors also used silica columns, they used them after successive washes and triphasic precipitation based on chloroform and alcohols. The combination of CTAB and columns has been used often, but always in combination with liquid nitrogen, phenol chloroform, and isoamyl alcohol. This procedure skips those steps, as well as the use of reagents that are sometimes difficult to access.

Amplification by RAPDs and ISSR markers produced clear and sharp bands, demonstrating that the extraction protocol proposed in this study produces high-quality DNA

## Figures and Tables

**Table 1:** Ecotypes of *Gossypium barbadense* L. UCV arboretum.

Code	Fiber Color	Varietal Type	Specie
GB01	Cream	Native colored cotton	*Gossypium barbadense* L. subsp peruano
GB02	Fifo	Native colored cotton	*Gossypium barbadense* L. subsp peruano
GB03	Ligtht brown	Native colored cotton	*Gossypium barbadense* L. subsp peruano
GB04	Dark brown	Native colored cotton	*Gossypium barbadense* L. subsp peruano
GB05	Orange-brown	Native colored cotton	*Gossypium barbadense* L. subsp peruano
GB06	Reddish	Native colored cotton	*Gossypium barbadense* L. subsp peruano
GB07	Fine red	Native colored cotton	*Gossypium barbadense* L. subsp peruano
GB08	White	Native colored cotton	*Gossypium barbadense* L. subsp peruano

**Table 2:** CTAB solution.

Extraction Buffer 01 (Extraction Solution)	Extraction Buffer 02 (Precipitation Solution)
2% (*w*/*v*) CTAB	1% (*w*/*v*) CTAB
100 mM Tris HCl (pH 8.0)	50 mM Tris HCl (pH 8.0)
20 mM EDTA (pH 8.0)	10 mM EDTA (pH 8.0)
1.4 M NaCl	1% PVP

**Table 3:** Sequence of primers.

Primers	Sequence
V-ANR-F	GGACTAGTGCTTGTGTCGTGGGTGGCA
V-ANR-R	TTGGCGCGCCTGCGGCAGATGATGTCAAGA
V-CHS-F	GGACTAGTGCACAAAACCGAGTTGAAAG
V-CHS-R	TTGGCGCGCCGGTCCACGAAAGGTAACAGCA
V-LAR-F	GGACTAGTCCAACGTATATCTTAGTCCGCTCT
LAR-R	TTGGCGCGCCCTCCTAATCTTGCTCTTTTGCT

Source: [30]

**Table 4:** Spectrophotometric analysis using a Nano Drop ND-2000.

Cultivar Code	Fiber Color	Concentration of DNA	260/280 Ratio	260/230 Ratio
Gb01	Cream	241.5	2.19	3.14
Gb02	Fifo	233	2.18	3.03
Gb03	Light brown	504	2.18	1.94
Gb04	Dark brown	454	2.15	2.26
Gb05	Orange-brown	177	2.14	2.80
Gb06	Reddish	147	2.14	2.44
Gb07	Fine red	264	2.14	1.8
Gb08	Brown	520.8	2.18	1.97

## Data Availability

The datasets analyzed during the current study are available from the corresponding author upon reasonable request.
